# Responding to the challenges of Global Children Surgery: a unique program in Burkina Faso

**DOI:** 10.1007/s00383-024-05903-w

**Published:** 2024-11-15

**Authors:** Sophie Inglin, Anata Bara, Yacouba Traoré, Saïd N. Ganame, Abou Coulibaly, Bertille Ki, Seydou Barro, Karim Barro, Emile Bandre, Seni Kouanda, Barbara E. Wildhaber

**Affiliations:** 1 Institute of Global Health, University of Geneva, Geneva, Switzerland; 2 Division of Pediatric and Adolescent Surgery, Unit of Pediatric Surgery, Department of Pediatrics, Gynecology, and Obstetrics, University Hospitals of Geneva, Rue Willy Donzé 6, 1205 Geneva, Switzerland; 3 Ministry of Health and Public Hygiene, Ouagadougou, Burkina Faso; 4 Division of Pediatric Surgery, University Hospital of Sourou Sanou, Bobo-Dioulasso, Burkina Faso; 5 Biomedical and Public Health Department, Research Institute of Health Sciences, National Center for Scientific and Technological Research, Ouagadougou, Burkina Faso; 6 Division of Anesthesia and Intensive Care, Pediatric University Hospital of Charles de Gaulle, Ouagadougou, Burkina Faso; 7 General Direction, University Hospital of Sourou Sanou, Bobo-Dioulasso, Burkina Faso

**Keywords:** Pediatric surgery, Low-to-middle income countries, Global surgery, Health system strengthening approach

## Abstract

**Purpose:**

The challenges are immense when it comes to enhancing the development of pediatric surgery in low- and middle-income countries in line with current recommendations for holistic and sustainable approaches. The Pediatric surgery development plan in Burkina Faso was started in 2020. This paper reports on our unique experience, focusing on the main actions and indicators monitored.

**Methods:**

The program was developed based on the core principles of the Global Initiative for Children's Surgery, employing a comprehensive health system-strengthening strategy. Our approach aimed to address the pediatric surgical ecosystem through partnerships, research, and interventions at all levels of the healthcare system.

**Results:**

Significant actions were implemented across various domains, including infrastructure, quality of care, workforce, community awareness, research, and advocacy. These efforts have led to a substantial strengthening of the pediatric surgical ecosystem in the country, achieving major milestones and outcomes in each of these areas.

**Conclusion:**

This program has not only helped to create a major impulse for the expansion of pediatric surgery in Burkina Faso, but also enriched the community of interest with a robust implementation program to confirm the main challenge to succeed: integrating this most essential discipline into the wider framework of global health.

## Introduction

In 2017, it was estimated that up to 67% of children worldwide lack safe, affordable, and timely surgical care, and among the 1.1 billion children and adolescents without access to such care, 65% live in lower-middle-income countries (LMICs) [1]. This is particularly significant given that children make up to 50% of the population in LMIC [2]. As for their surgical needs, Butler et al. showed in 2015 in a cluster randomized cross-sectional household survey that 19% of children in these countries had a surgically correctable problem that was considered to need a surgical consultation, yet almost two-thirds of them had not received surgical care to correct it [3]. Sub-Saharan Africa (SSA) exhibits the greatest needs in pediatric surgery [4], with surgical conditions accounting for between 6 and 12% of all pediatric admissions, highlighting the critical demand for improved access to surgical care [5].

While pediatric surgery has received growing attention from SSA governments, significant challenges remain, such as a lack of human resources, inadequate infrastructure, and financial barriers, indicating that further investment and action are still necessary to effectively upscale pediatric surgical services [6].

In Burkina Faso, one of the SSA countries in West Africa, pediatric surgery also faces many barriers. To provide context, in 2018, during the diagnostic phase of the program described here, Burkina Faso had a total population of approximately 20.3 million, including around 9.5 million children under the age of 15 years. At that time, only six pediatric surgeons were practicing in the country, representing one pediatric surgeon for about 1.58 million children. Further, only six anesthetists were trained and qualified to treat children. The pediatric university hospital Charles de Gaulle of Ouagadougou was the country’s one and only medical facility equipped to care for children requiring surgery. Its occupancy rate was 107% and over 2000 children were constantly on the waiting list (unpublished data).

Health system-strengthening approaches are definitely needed to develop pediatric surgery as an essential component of global health. Thus, in the recent years, a plea was made to develop pediatric surgery by considering the entire ecosystem in which it is embedded. As a consequence, the Global Initiative for Children’s Surgery (GICS) community identified i) training and staffing, ii) physical resources, iii) quality and safety, and iv) research as thematic areas that need to be addressed to improve children's surgery globally [7]. Indeed, these thematic areas represent subsets of the WHO’s health systems’ building blocks that collectively contribute to a well-functioning health system [8].

Pediatric surgery has come a long way in the past decade, and many research articles have shed light on the issues at stake, particularly in SSA. But in reality, too few holistic programs have been conducted and successfully translated into implementation, as recently stated by Emmanuel Ameh: “Until now most initiatives and efforts have been focused on identifying the problems and challenges in LMICs, without much attention given to implementation of initiatives that actually changes the on the ground situation in a coordinated and incremental manner” [9].

In this context, our program entitled *Pediatric surgery development plan in Burkina Faso* was designed to respond to the challenge of an integrated approach to improve the pediatric surgical ecosystem, promoting the principles of the health system-strengthening approach. The aim of this medical and social program is to bring a holistic and sustainable response, by targeting specific priority actions at all levels of the healthcare system. This paper presents our experience, reporting on the first 4 years of the program and focusing on the main results and monitored indicators.

## Methods: design and implementation process

This program followed all the stages of project cycle management. Table 1 highlights the various methodological processes we used in the four major stages of the project cycle: (i) identification, (ii) preparation, (iii) implementation, and (iv) evaluation. During the identification stage, the required needs were defined through a series of workshops and interviews using the WHO’s health systems building blocks framework to brainstorm, with key experts of Burkina Faso, on how to improve the performance of the pediatric surgical ecosystem (workforce, service delivery, medical products, vaccines and technologies, financing, leadership and governance, and information systems). During a 1-year preparation phase, a multidisciplinary team from Switzerland and Burkina Faso designed a comprehensive program grounded in the latest international recommendations and aligned with the core pillars of the Global Initiative for Children's Surgery [9]. The program's intervention areas, specific goals, actions, and processes are detailed in Table 2, reflecting the global health philosophy emphasized in previous phases. Throughout the entire program, oversight was provided within the framework of a collaboration agreement between the Geneva University Hospitals, the Burkina Faso Ministry of Health, and the two partner hospitals in Ouagadougou and Bobo-Dioulasso. The total operating cost amounted to approximately 1.5 million Swiss francs.

**Table 1:** Major methodological processes which were followed for the different stages of the program

Stage	Duration	Methodological process	Outputs
Identification	3 months	Consultation meetings to discuss the challenges in providing optimal surgical care for children in Burkina Faso with 40 experts Literature review: gray literature (government policies, manuals, etc.)	Report of all collected data organized according to the WHO health system building blocks framework Problem tree Strategic overview
Preparation	1 year	Interviews with key stakeholders Data collection from various channels (data from hospitals, Ministry of Health, etc.)	Design of action plan (Table 2) Logical framework Planification
Negotiation	1 year	Meetings with the Ministry of Health and related departments	Memorandum of understanding Convention of collaboration
Implementation	4 years	Action research (quantitative) Data collection and analysis Reporting and recommendations	Monitoring of indicators (Table 3) Operational reports
Monitoring and evaluation

**Table 2:** Summary of the 4 years program areas of interventions, their specific goals, led actions, and the main processes used

Areas of intervention	Level of action	Specific goals	Actions	Main processes
Infrastructure	Regional	To build infrastructure capacity for safe, timely, and quality pediatric surgical and anesthetic care in UH of Bobo-Dioulasso (CHUSS)	Sanitation operation Renovation and construction work Supply of equipment Implementation of a computerized maintenance management system software with reproductible guidelines (CMMS) Training of all related hospital’s services regarding CMMS Creation of a procedure manual on CMMS Updating of a hospital maintenance plan Advocacy to increase the workforce	Site diagnosis by biomedical engineers Setting up of a steering committee for construction work progress tracking Creation of plans and cross-checking by experts Analysis of the maintenance service Reorganization of the maintenance service
Quality of care	Regional	To strengthen the care standardization process in two UH (Charles de Gaulle in Ouagadougou, CHUSS)	Creation of a clinical database with clinical quality indicators Implementation of quality tools and measures Creation of clinical protocols in the field of surgical care	Articulation of objectives by discipline Hands-on collaboration with teams from Geneva hospitals
Workforce	National	To enhance the skills of personnel involved in pediatric surgical and anesthetic care To optimize the sustainability of the different trainings	Creation of five training modules dedicated to surgical pediatrics On-site short trainings for health professionals Sustainability plans: integration of pediatric surgical teaching units into national nursing training programs/training of trainers approach	Workshops with medical and paramedical professionals Workshops with the National school of public health Pre- and post-test evaluations Iterative improvement
Community awareness	Regional	To strengthen the collaboration and awareness among traditional healers and families	Training of community health workers Awareness raising activities within communities through educational talks and radio broadcasts	Weekly planning of village outings by the field supervision team and community-based health workers Monthly meetings to report and validate data to the district management team and to the regional health direction Transmission of validated data (monthly report)
	National	To provide the country with reproducible awareness raising and educational tools	Creation of an image box on domestic accidents validated by the Ministry of Health for community awareness purpose Creation of a pocket booklet on fractures for traditional healers validated by the country's scholarly societies Creation of a guidebook for optimal detection of congenital anomalies at the first level of care	Interview and workshops with various audiences National workshops of validation
Research	National	To strengthen epidemiological and clinical research in pediatric surgery	Nationwide survey of pediatric surgical capacity to obtain an up-to-date map of the current situation in all the country’s hospitals Study on prevalence and determinants of domestic accidents among children aged 0 to 14 years in the health district of Orodara, Burkina Faso Study on determinants of mothers' knowledge of emergency actions and attitudes in domestic accidents involving children in the health district of Orodara, Burkina Faso Study on pre-interventional incidence of structural congenital malformations in live births in the city of Bobo Dioulasso, Burkina Faso	Protocol drafting Submission to ethics committee Data collection and analysis Data dissemination
Advocacy	National	To increase the awareness of pediatric surgery by capitalizing on the program as an advocacy tool To promote advocacy for pediatric surgery to be considered as per se as in the national health strategy (versus “surgery” only)	Review of the national surgery and anesthesia strategy Collaboration with the Ministry of Health's Department of Care Quality and Safety Elaboration of a roadmap in line with recommendations issued by OReCs (optimal resources for children's surgical care)	Stakeholder mapping Meetings with stakeholders at the national level Ongoing program capitalization Large data collection of the pediatric surgical capacity in all relevant hospitals in the country

## Results

The numerous interventions of the program were implemented as part of the *Pediatric surgery development plan in Burkina Faso*. Table 3 highlights the achieved results by presenting the various monitoring indicators.

**Table 3:** Data before and after the development team’s interventions. UH, university hospital

Objectives	Pre-intervention (year 2018)	Post-intervention (year 2023)
*Infrastructure, equipment, and maintenance (in UH Bobo-Dioulasso)*		
Operating theaters	4 shared operating theaters where both, adults and children are operated	Five operating theaters including one dedicated to pediatric surgery
Infrastructure	28-bed hospital wing	44-bed hospital wing
Equipment	Restricted equipment	40 environment-adapted pediatric biomedical equipment for optimal functioning the operating theater and wards
Maintenance	Non-existing maintenance system	700 equipment has been collected, sorted, and recycled 1 hospital maintenance plan created 1000 equipment inventoried using a computerized maintenance management system software (CMMS) 5 staff trained in CMMS and 25 departments sensitized
Pediatric surgery Personnel	1 pediatric surgeon 5 anesthesiologists 0 scrub nurses 8 ward nurses	5 pediatric surgeons 5 anesthesiologists trained as trainers in pediatric anesthesia 8 scrub nurses 23 ward nurses
Patients	849 children admitted to surgery	1327 children admitted to surgery
*Quality of care (in UH Bobo-Dioulasso and UHCDG)*		
Quality measures and clinical database	No systematic use and monitoring of quality measures of the UH of Bobo-Dioulasso	Use of the following tools (monthly rate for 12/2023): 73% for surgical checklist 52% for swab count Implementation of informed consent form for parents, admission document before hospitalization, monitoring form, written post-operative instructions 25 indicators collected monthly (number of operations, types of operation, lengths of hospital stay, etc.) 18 additional care protocols (ongoing)
*Workforce (nationwide)*		
Pediatric surgeons	11 pediatric surgeons	25 pediatric surgeons trained in specific pathologies through various training modules
Anesthetists and nurse anesthetists	6 anesthetists and 0 nurse anesthetists trained in pediatric care	136 anesthesiologists and nurse anesthetists trained in pediatric care through the course *Safer Anesthesia* from Education (*SAFE*)
Scrub nurses	0 scrub nurses trained in pediatric care	95 scrub nurses trained in pediatric surgical care through a new on-site training module
Ward nurses	0 nurses trained in pediatric surgical care	350 nurses trained in pediatric surgical care through a new on-site training module
Midwives	0 midwives trained in congenital malformations	310 midwives trained in congenital malformations through a new on-site training module
*Community awareness (nationwide with pilots at the regional level)*		
Traditional healers	No traditional healers trained in pediatric fracture and burn management	700 traditional healers sensitized in pediatric fracture and burn management
Families	No existing projects on domestic accidents in rural areas among children	212 trained community health workers in domestic accidents 100′000 people sensitized in rural areas
*Research*		
Data and communication	Few data on pediatric surgery at the national level	4 scientific studies, co-managed by the Geneva University Hospitals and the Ouagadougou Institute for Health Research 6 abstracts and oral communications at 3 pediatric surgery conferences 3 master's and thesis projects
*Advocacy for pediatric surgery (nationwide)*		
Policy	No baseline assessment of the hospital surgical capacities in the country	160 indicators on the hospital surgical capacities analyzed 10 meetings with the Ministry of Health and communities of concern 7 communications through formal national media (TV, print media, radio) 20 additional pediatric surgeons to be recruited by 2025 within the advanced pediatric surgery diploma program

### Infrastructure

To strengthen Burkina Faso’s pediatric surgery infrastructure and to provide the country with a second technical platform for high-quality pediatric surgical and anesthetic care, we decided to focus on the University Hospital Souro Sanou (UHSS) in Bobo-Dioulasso, a city in the western part of Burkina Faso. This region is home to 28% of the country’s children aged 0–14 years. This choice was oriented by Burkinabé experts during the needs assessment stage and was intended not only to relieve congestion in the capital, but also to reduce the influx of people from the south-west to the center for any child requiring pediatric surgical care.

The rehabilitation work, including a pediatric operating theatre and a recovery room, as well as the construction of a new hospitalization wing, have doubled the hospital's capacity and enabled the team to treat more complex pathologies. In line with hospital statistics, 849 children were admitted to pediatric surgery in 2018, compared with 1327 by the end of 2023 (Table 3). Since then, hundreds of operations have been carried out, relieving congestion at Ouagadougou's Children's Referral Hospital (UHCDG). Indeed, in June 2024, the UHCDG evacuated children requiring treatment for posterior urethral valves to the UHSS and confirmed the importance of the UHSS as the second reference center in this field, now equipped with the necessary technical facilities and skills.

These accomplishments were achieved through interventions such as a hospital sanitation operation, the acquisition of biomedical equipment, and the implementation of a computer-based sustainable maintenance system to ensure the longevity of the new equipment and, more broadly, the entire UHSS equipment pool.

### Quality of care

An important component of this program was the objective to improve the quality of pre-, per- and post-operative care in the two university hospitals of the country: the partner hospital UHSS and the UHCDG in Ouagadougou. The former is the beneficiary of the new infrastructure and was therefore in full development, while the latter has long been a reference center in the field of pediatric surgery, located in the capital of the country. As part of the UHSS partnership, the Swiss–Burkinabe teams, through an interdisciplinary collaboration, have helped to create a common dynamic and introduced a number of clinical tools. These included the implementation of (i) the surgical checklist, (ii) gauze count in the operating room, (iii) structured pain assessment on the ward using the Evendol technique [10], (iv) regular monitoring of fluid balance, (v) structured admission and discharge notes, and (vi) a nursing monitoring sheet to provide the clinical team with a systematic point-by-point approach to monitor the patient and his equipment, all through hands-on collaboration. Quality indicators were recorded monthly by a reference person of the pediatric surgery division and shared with the hospital's general manager to better monitor and follow the quality progress of the discipline (Table 3). The average utilization rate of the surgical checklist was 80% during the last year and the one related to the swab count sheet was 65%. In both hospitals, the development and drafting of care protocols proved to be a priority, such as patient preparation process for laparoscopy, the pull-through operation in Hirschprung's disease, bone surgery, and the skin graft dressing protocol for burned children.

This entire work was promoted through clinical research and presentations at pediatric surgery meetings in the country and accross the international region.

### Workforce

As part of this program, significant needs were identified in staff capacity building, particularly to enhance the skills of practicing healthcare professionals through on-site training opportunities. Training modules lasted between 3–5 days and were delivered by a pool of local trainers. Various fields were targeted such as (i) pediatric surgery, (ii) pediatric anesthesia, (iii) pre- and post-operative patient care, (iv), operating room management, and (v) congenital anomalies. As shown in Table 3, hundreds of professionals, from university and regional hospitals, have benefited from skills enhancement in surgical pediatrics over the last 4 years. We describe below the seminal outputs related to the first three training modules.

First, in terms of training in pediatric surgery, the advanced diploma program in Burkina Faso has been in place for several years (2017). As part of our development program, we have strengthened this curriculum by adding two annual master classes for physicians seeking specialization in pediatric surgery. Originally held exclusively in the capital, these sessions are now offered at two hospitals, with the newly renovated UHSS serving as a primary training site. Each week-long course focuses on specific topics such as cleft lip and palate, anorectal malformations, and Hirschsprung’s disease. The program features videoconferences with experts, hands-on workshops using mannequins, and lectures from senior local pediatric surgeons. Secondly, at the time of inception in 2018, only six anesthesiologists had received training in children’s care, compared to 36 after our intervention (representing 50% of all national anesthesiologists). They benefitted from the *Safer Anesthesia From Education* (SAFE) course, a specialized training program designed to improve the skills and practices of pediatric anesthesiologists, based on practice and theory and adapted to local needs and resources, incorporating the train-the-trainer approach. Another benefit of the SAFE methodology is that trainers enabled anesthesiologists and nurse anesthetists from *regional* hospitals to train colleagues from *tertiary* hospitals. Thirdly, nurses' skills have been significantly improved through two key methods. On one hand, on-site training was provided by a team of Burkinabé trainers, using a newly designed training module tailored to local needs and realities. This pilot program, conducted over 4 years, included pre- and post-assessments, as well as evaluations from participants. The training specifically targeted nurses involved in the surgical care of children within hospitals, who had not previously received specialized training in pediatric care. On the other hand, to enhance the skills of future nurses, a crucial focus was placed on pre-graduate students and their basic curriculum. The National School of Public Health spearheaded this effort by conducting a series of workshops where experts reviewed and revamped the pediatric surgery curriculum. They identified key gaps and developed new teaching modules, ensuring that all 500 new students annually will be thoroughly prepared in peri- and post-operative pediatric care. This significant step represents a major advancement for the country and promises to substantially improve the quality of care.

### Community awareness

It is crucial to include the community when implementing pediatric surgery initiatives. Following our identification phase, we recognized that the country’s pediatric surgery divisions were overloaded with pediatric cases presenting with complex bone fractures and burns. Two projects were therefore deployed to raise awareness of these important community health issues among families and traditional healers alike. Firstly, we developed a training course for traditional healers to highlight the risks associated with open fractures and the critical need for early referral. This training, delivered by two pediatric surgeons from Burkina Faso, reached 700 practitioners and was assessed after each session. Participants appreciated the use of a notebook to reinforce key messages and ensure ongoing awareness. The second project aimed to prevent domestic accidents through a pilot initiative, which was informed by a prevalence study of domestic accidents in rural areas. The project sought to integrate this activity into the long-term minimum package of services for community-based health workers. An image box dedicated to educational talks in the villages was created in a participatory manner. This was followed by the training of community-based health workers to deliver effective awareness-raising in the targeted villages, reaching nearly 100,000 people through educational talks and radio broadcasts. Table 2 shows the detailed our activities in community awareness and Table 3 highlights the number of beneficiaries we were able to reach.

### Research

The different actions of this program have benefited from ongoing data collection by collaborating with the Research Institute of Health Sciences, a Burkinabe research institute located in the capital. We carried out a number of research action projects, which all have enabled us to better orientate our interventions and support our advocacy work. The list of carried out studies is shown in Table 3.

### Advocacy

As part of the “World Alliance for Safer surgery to save lives” launched by the WHO [11], Burkina Faso adopted in 2021 a national strategy and strengthening plan 2021–2025 for surgical and anesthetic care. As part of this strategy, an assessment of general surgery was carried out, but no data specific to pediatric surgery was made available [12]. One of the most important interventions of our program has been to advocate among health authorities to ensure that pediatric surgery would be considered per se in the near future. A number of milestones have been reached, in collaboration with the Ministry of Health's Department of Care Quality and Safety, through data collection, meetings and advocacy, stakeholder mobilization, use of the media, and networking (Table 3). The program as a whole has led to a greater visibility and consideration of this discipline. By way of example, the Council of Ministries decided in March 2024 to promote the strengthening of human resources in pediatric surgery, with more than 20 pediatric surgeons to be recruited by 2025. This commitment has already become a reality as evidenced by the current enrollment of over 26 new candidates in the country’s advanced diploma in pediatric surgery.

## Discussion

As noted by many experts, great efforts have been invested in global children's surgery in LMIC, but these changes are recent and still slow [13]. For too long, pediatric surgery has been considered as an expensive and a non-essential service [14]. However, the provision of surgical healthcare is a foundation of treatment needed to manage approximately one-third of the global burden of disease [15]. Reinforcing the pediatric surgery ecosystem in LMIC is thus key to attain the UN’s Sustainable Development Goal 3 [16].

Our program, *Pediatric surgery development plan in Burkina* Faso, was designed to increase pediatric surgical capacity in the country, but more generally to highlight the importance of targeted actions in line with a *holistic, ecosystem-based intervention approach*, including the development of infrastructure, equipment, quality of care, training, research, advocacy, and community work. Indeed, only a few experts around the world contribute to the delivery of a global children's surgery that meets the requirements of a sustainable development. An example is “KidsOR”, a non-governmental organization based in Scotland which promotes a global health perspective in the field of pediatric surgery [17].

Generally, and according to Dare et al., three main themes tend to influence the prioritization of surgery as a health issue at the national level including (1) the degree of sustained and effective domestic advocacy by the local surgical community, (2) the national political and economic environment in which advocacy efforts take place, and (3) the influence of international actors on agenda setting [18]. Our Pediatric surgery development plan in Burkina Faso, underpinned by a collaboration agreement with hospitals and the country’s Ministry of Health, and financially supported by numerous international and local sponsors and partners, has made it possible to commit multiple resources, interventions, and future perspectives to increase advocacy at a political level. This program was intended to bring together all those involved in pediatric surgery and anesthesia, including the Burkinabe Society of Pediatric Surgery and the Society of Anesthesia, Intensive Care and Emergency Medicine, to increase their voice in favor of the development of children's surgery.

Finally, we wish to emphasize the importance of community work and community health in pediatric surgery. Although pediatric surgery is frequently viewed as a highly technical specialty, it encompasses essential aspects such as prevention, disease recognition, and first aid measures. Our program has highlighted the critical importance of integrating a community health approach to address various barriers effectively and to involve families and traditional healers in the process.

Despite being holistic, it was impossible to act on every barrier. Indeed, we were unable to act on the financial barrier. As showed by Yap et al. in their recent study from 2023, a full 40% of families of children in SSA who undergo surgery incur catastrophic healthcare expenditure, shouldering economic consequences such as forfeited wages and debt [19]. But of note, from 2016, in Burkina Faso there has been a system of free care for pregnant women and children under 5 years old, comprising surgical conditions for which curative treatment is available within health centers. This also includes free transport for children under 5 years. This policy is a real asset in the country, but still raises fundamental ethical issues in the care of children over 5 years. Furthermore, it must be mentioned that the socio-political situation the country has been experiencing for several years is interfering with the health governance, in favor of other security-related priorities. However, the interest and commitment of the main authorities in our actions to promote pediatric surgery has been very encouraging and does not appear to have been immediately affected by this issue.

In conclusion, there is a pressing need for more holistic pediatric surgery development programs. The unique experience gained in Burkina Faso has demonstrated that an ecosystemic program, grounded in the latest international recommendations, and adhering to a health systems strengthening approach, can lead to significant improvements in the country's pediatric surgical capacity and enhance its recognition by national health authorities. This program has not only helped to create a major impulse for the development of this discipline, but also provides the community of interest with a concrete implementation program to overcome the main challenge we face: integrating pediatric surgery into the wider framework of global health.

## Data Availability

No datasets were generated or analysed during the current study.
